# Nuclear Respiratory Factor 1 Negatively Regulates the P1 Promoter of the Peroxisome Proliferator-Activated Receptor-γ Gene and Inhibits Chicken Adipogenesis

**DOI:** 10.3389/fphys.2018.01823

**Published:** 2018-12-19

**Authors:** Tingting Cui, Tianyu Xing, Jiaxin Huang, Fang Mu, Yanfei Jin, Xin You, Yankai Chu, Hui Li, Ning Wang

**Affiliations:** ^1^Key Laboratory of Chicken Genetics and Breeding, Ministry of Agriculture and Rural Affairs, Harbin, China; ^2^Key Laboratory of Animal Genetics, Breeding and Reproduction, Education Department of Heilongjiang Province, Harbin, China; ^3^College of Animal Science and Technology, Northeast Agricultural University, Harbin, China; ^4^Institute of Animal Science of Heilongjiang Province, Qiqihar, China

**Keywords:** nuclear respiratory factor 1, chicken, *PPARγ*, transcription, adipogenesis

## Abstract

Peroxisome proliferator-activated receptor-γ (PPARγ) is a master regulator of adipogenesis, and alterations in its function are associated with various pathological processes related to metabolic syndrome. Recently, we found that the chicken *PPARγ* gene is regulated by three alternative promoters (P1, P2 and P3), producing five different transcript isoforms and two protein isoforms. In this study, the P1 promoter structure was characterized. Bioinformatics identified six putative nuclear respiratory factor 1 (NRF1) binding sites in the P1 promoter, and a reporter assay showed that NRF1 inhibited the activity of the P1 promoter. Of the six putative NRF1 binding sites, individual mutations of three of them abolished the inhibitory effect of NRF1 on P1 promoter activity. Furthermore, a ChIP assay indicated that NRF1 directly bound to the P1 promoter, and real-time quantitative RT-PCR analysis showed that *NRF1* mRNA expression was negatively correlated with *PPARγ1* expression (Pearson’s *r* = -0.148, *p* = 0.033). Further study showed that NRF1 overexpression inhibited the differentiation of the immortalized chicken preadipocyte cell line (ICP1), which was accompanied by reduced *PPARγ1* mRNA expression. Taken together, our findings indicated that NRF1 directly negatively regulates the P1 promoter of the chicken *PPARγ* gene and inhibits adipogenesis.

## Introduction

Adipogenesis plays central roles in energy homeostasis and is significantly associated with obesity. Adipogenesis is tightly controlled by a complex network of transcriptional regulators (Rosen et al., 2002; Garinshkolnik et al., 2014). PPARγ is a master regulator of adipogenesis and regulates the transcription of various genes involved in adipocyte differentiation and lipid accumulation (de la Rosa Rodriguez and Kersten, 2017; Kim et al., 2017; Park et al., 2017). Ectopic expression of PPARγ is sufficient to induce adipocyte differentiation in fibroblasts, and no factor has been reported to promote adipogenesis in the absence of PPARγ (Lee and Ge, 2014).

In mammals, the *PPARγ* gene is regulated by multiple alternative promoters. Because of alternative promoter usage and alternative splicing, the *PPARγ* gene produces various transcript isoforms, encoding two different protein isoforms: PPARγ1 and PPARγ2 (Zhu et al., 1995; Lee and Ge, 2014; Chandra et al., 2017). The human *PPARγ* gene has four alternative promoters designated the PPARγ1, PPARγ2, PPARγ3 and PPARγ4 promoters while the mouse *PPARγ* gene has two alternative promoters designated the PPARγ1 and PPARγ2 promoters (Zhu et al., 1995; Al-Shali et al., 2004; Lee and Ge, 2014; Chandra et al., 2017). To date, a number of transcription factors and coregulators have been identified that regulate the alternative promoters of the *PPARγ* gene in humans and mice. For example, E2F transcription factor 1 (E2F1), early B-cell factor 1 (EBF1), and sterol regulatory element-binding protein-1 (SREBP1) directly bind to mouse PPARγ1 promoter and enhance the expression of the *PPARγ1* transcript (Lee and Ge, 2014). Nuclear factor E2-related factor 2 (NRF2), Krüppel-like factors 5 (KLF5), KLF9 and KLF15 directly bind to mouse PPARγ2 promoter and enhance the expression of the *PPARγ2* transcript, while KLF2 directly binds to mouse PPARγ2 promoter and decreases *PPARγ2* transcript expression (Pi et al., 2010; Lee and Ge, 2014). Forkhead box class O1 (FOXO1) can directly bind to and reduce human PPARγ2 promoter activity, and in addition, it can indirectly inhibit human PPARγ1 promoter activity (Armoni et al., 2006).

Recently, we demonstrated for the first time that the chicken *PPARγ* gene is regulated by three alternative promoters designated P1, P2 and P3, producing five different transcript isoforms (*PPARγs 1-5*) due to alternative splicing and promoter usage (Duan et al., 2015). *PPARγ1* initiates from the P1 promoter, *PPARγs 2-4* from the P2 promoter and *PPARγ5* from the P3 promoter. Among these five different transcript isoforms, *PPARγ1* is highly expressed in various chicken tissues, including adipose tissue, liver, kidney, spleen and duodenum (Duan et al., 2015). The transcriptional regulation of the P1 promoter is still unknown. In the present study, we characterized the P1 promoter organization and demonstrated that nuclear respiratory factor 1 (NRF1) negatively regulates the P1 promoter of the *PPARγ* gene and inhibits chicken adipogenesis.

## Materials and Methods

### Ethics Statement

The research project was approved by the Institutional Biosafety Committee of Northeast Agricultural University (Harbin, P. R. China). Plasmid construction and transfection were performed in accordance with the guidelines for the Regulation on Safety Administration of Agricultural Genetically Modified Organisms (RSAGMO) established by the People’s Republic of China (Revised version 2017).

### Tissues

The abdominal fat tissue samples used in this study were from a previous study by our group (Zhang et al., 2017). These samples were obtained from generation 19 of Northeast Agricultural University broiler lines divergently selected for abdominal fat content (NEAUHLF) and composed of a total of 70 abdominal fat tissues of male birds (five birds per line and per time point) from 1 to 7 weeks of age. These samples were stored in liquid nitrogen until total RNA extraction.

### Plasmid Construction

The chicken *PPARγ* P1 promoter and its subsequent 5′-truncation mutants were generated by PCR from chicken genomic DNA using the designated forward primers and a common reverse primer (Cloning P1+108 promoter R) as shown in Table 1 and then subcloned into the promoterless luciferase expression vector pGL3-Basic (Promega, United States). Site-directed mutagenesis was performed by DNA synthesis (GENEWIZ, China). The site-mutated P1 promoters were cloned into a pGL3-Basic vector. For the NRF1 overexpression vector, the full-length coding region of chicken *NRF1* was amplified by RT-PCR from chicken abdominal fat tissue total RNA and cloned into an empty pCMV-HA vector (Clontech, United States). All primers are shown in Table 1, and all final constructs were confirmed by DNA sequencing.

**Table 1:** PCR primers used in this study.

Primer name	Sequence
Cloning P1-1891 promoter F	F:5′-ATTTCTCTATCGATAGGTACCTCCTGCCTCAATCTGCTAAAATA-3′
Cloning P1-950 promoter F	F:5′-ATTTCTCTATCGATAGGTACCGTAATTTGATGTATTCAACTCTAATCTATAA-3′
Cloning P1-450 promoter F	F:5′-ATTTCTCTATCGATAGGTACCGCGACTACACGCAGCGCA-3′
Cloning P1-327 promoter F	F:5′-ATTTCTCTATCGATAGGTACCCCAGATCCACTCCAGGGC-3′
Cloning P1-231 promoter F	F:5′-ATTTCTCTATCGATAGGTACCGAACAGATTGGGTCTAAAGGG-3′
Cloning P1+1 promoter F	F:5′-ATTTCTCTATCGATAGGTACCGGCGGTGCCCCGGCGGGG-3′
Cloning P1+108 promoter R	R:5′-CAGTACCGGAATGCCAAGCTTTGGCGCTGTCAAGTCTCA-3′
Cloning full-length *NRF1* CDS	F: 5′-TGGCCATGGAGGCCCGAATTCGGATGGAAGAACACGGCGTG-3′
	R: 5′-GATCCCCGCGGCCGCGGTACCTCACTGTTCCAAAGTTACCA-3′
qRT-PCR *NRF1*	F: 5′-ACCCATCCATCCGTAAGAG-3′
	R: 5′-CTTGCGTACCACATTCTCC-3′
qRT-PCR *PPARγ1*	F: 5′-GGAGTTTATCCCACCAGAAG-3′
	R: 5′-AATCAACAGTGGTAAATGGC-3′
qRT-PCR *NONO*	F: 5′-AGAAGCAGCAGCAAGAAC-3′
	R: 5′-TCCTCCATCCTCCTCAGT-3′
qRT-PCR *FABP4*	F: 5′-ATGTGCGACCAGTTTGT-3′
	R: 5′-TCACCATTGATGCTGATAG-3′
qRT-PCR *G0S2*	F: 5′-GACGGCAAGGATGGAAAAGAT-3′
	R: 5′-GTCGTAGTGGTTCTGCTCGTTGTA-3′
qRT-PCR *GPDH*	F: 5′-ACCTCCCATCCCATACCGA-3′
	R: 5′-CCACTCCACGCTGCCAACA-3′
qRT-PCR *AdipoQ*	F: 5′-GCAACAACAACGGGGTCT-3′
	R: 5′-AGGGGAATTTTCTGGTACATAG-3′
ChIP-qPCR P1 promoter	F: 5′-GAGCCCCGACCCGCGCAGCGCCCAC-3′
	R: 5′-ATAAACTCCCCGGGCCGGCCCATCC-3′
ChIP-qPCR Fluc	F: 5′-AAAACGGATTACCAGGGATTTCAGT-3′
	R: 5′-AGCGACACCTTTAGGCAGACCAGT-3′

### Cell Culture

DF1 cells, chicken stromal vascular fraction (SVF) cells and an immortalized chicken preadipocyte (ICP1) cell line (Wang et al., 2017) were maintained in Dulbecco’s-modified Eagle’s medium (DMEM) with high glucose (Gibco, United States) or DMEM: nutrient mixture F12 (DMEM/F12) (Gibco) supplemented with 10% fetal bovine serum (FBS) (BI, Germany) plus 100 units/ml penicillin and 100 units/ml streptomycin in a humidified incubator at 37°C and 5% CO_2_.

### Transfection and Luciferase Assays

After reaching 70–80% confluence, the DF1, ICP1 and SVF cells were washed with PBS, and transient transfections were performed using Lipofectamine 2000 reagent (Invitrogen, United States). A reporter luciferase assay was performed according to the manufacturer’s instructions with a Dual-Luciferase Reporter Assay System (Promega, United States) after 48 h of transfection. Firefly luciferase (*Fluc*) activity was normalized to Renilla luciferase (*Rluc*) activity.

### Western Blot Analysis

Cultured cells were washed three times with cold PBS and lysed in 6-well plates by using 100 μl RIPA Buffer (Beyotime Biotechnology, China) supplemented with 1 μl phenylmethanesulfonyl fluoride (Beyotime Biotechnology, China). Cell lysates were subjected to SDS-PAGE followed by Western blot analysis using an HA-specific antibody (Abmart, China) and a β-actin antibody (Beyotime Biotechnology, China). The blots were visualized using an ECL Plus detection kit (Beyotime Biotechnology, China).

### Chromatin Immunoprecipitation (ChIP) Assay

Chromatin immunoprecipitation was performed using a ChIP assay kit (Cell Signaling Technology, United States) as previously described (Funato et al., 2003; Ouyang et al., 2011; Deng et al., 2012). DF1 cells were cotransfected with pGL3P1-1891/+108 and pCMV-HA-NRF1 or an empty pCMV-HA vector for 48 h; 1 × 10^7^ transfected cells were then fixed with 1% formaldehyde at room temperature for 10 min and quenched in 125 mM glycine at room temperature for 5 min. The Chromatin was digested with 0.5 μl micrococcal nuclease into 100–900 bp DNA/protein fragments, and immunoprecipitated using with 5 μg of HA-specific antibody (Abcam, United States) or 5 μg of mouse IgG (Beyotime, China). The purified DNA fragments were analyzed by qPCR. The qPCR was performed on the 7500 Real-Time PCR System (Applied Biosystems, United States) with SYBR Green PCR Master Mix (Roche Molecular Systems, United States). A specific pair of primers was designed to detect the coding region of the firefly luciferase gene, which was used as a negative control. The primers used in the ChIP-qPCR assay are shown in Table 1. Non-immunoprecipitated DNA (2%) was used as an input control. ChIP qPCR data were normalized to input chromatin and then presented as fold enrichment over the negative control using the ΔΔCt equation (Tatler et al., 2016).

### ICP1 Cell Differentiation

Immortalized chicken preadipocyte cell lines were cultured to 50% confluence, and then, the cells were induced to differentiate by 160 μM sodium oleate (Sigma, United States). Subsequently, the medium was removed every 24 h and replaced with fresh medium containing DMEM/F12 containing 10% FBS together with 160 μM sodium oleate. Cells were harvested for RT-PCR every 24 h. Differentiation of ICP1 cells was continued for a total of 96 h.

### Oil Red O Staining

Oil red O staining was performed as described previously (Wang et al., 2017). Briefly, the differentiated ICP1 cells were washed twice with PBS and fixed by 4% paraformaldehyde for 30 min at 4°C, washed with PBS twice and distilled water twice, and stained with 0.5% Oil Red O staining solution in isopropanol and water (3:2) for 15 min at room temperature, followed by washing with PBS twice. Alternatively, for a quantitative assay, the stained cells were destained with isopropanol, and the optical density was determined at a 510 nm wavelength with a spectrophotometer (Ultrospec 1000, United Kingdom).

### RNA Isolation and Quantitative Real-Time RT-PCR

Total RNA was isolated using TRIzol reagent (Invitrogen, United States) following the manufacturer’s instructions, and cDNA was generated with a Transcriptor First Strand cDNA Synthesis kit (Roche Molecular Systems, United States), followed by analysis using a 7500 Real-Time PCR System (Applied Biosystems, United States) with SYBR Green PCR Master Mix (Roche Molecular Systems, United States). The primers used are shown in Table 1. Relative mRNA levels were normalized to the *NONO* gene (non-POU domain containing) using the 2^-ΔΔCt^ method.

### Statistical Analysis

Data are expressed as the mean ± SE of the mean. Student’s *t*-test was performed using Graph Pad Prism 5. Pearson’s *r* was used to determine the degree of correlation between *PPARγ1* and *NRF1* mRNA expression levels as described (Sun et al., 2014). Statistical significance was indicated by ^∗^*p* < 0.05, ^∗∗^*p* < 0.01.

## Results

### Characterization of the Chicken PPARγ P1 Promoter

**FIGURE 1:**
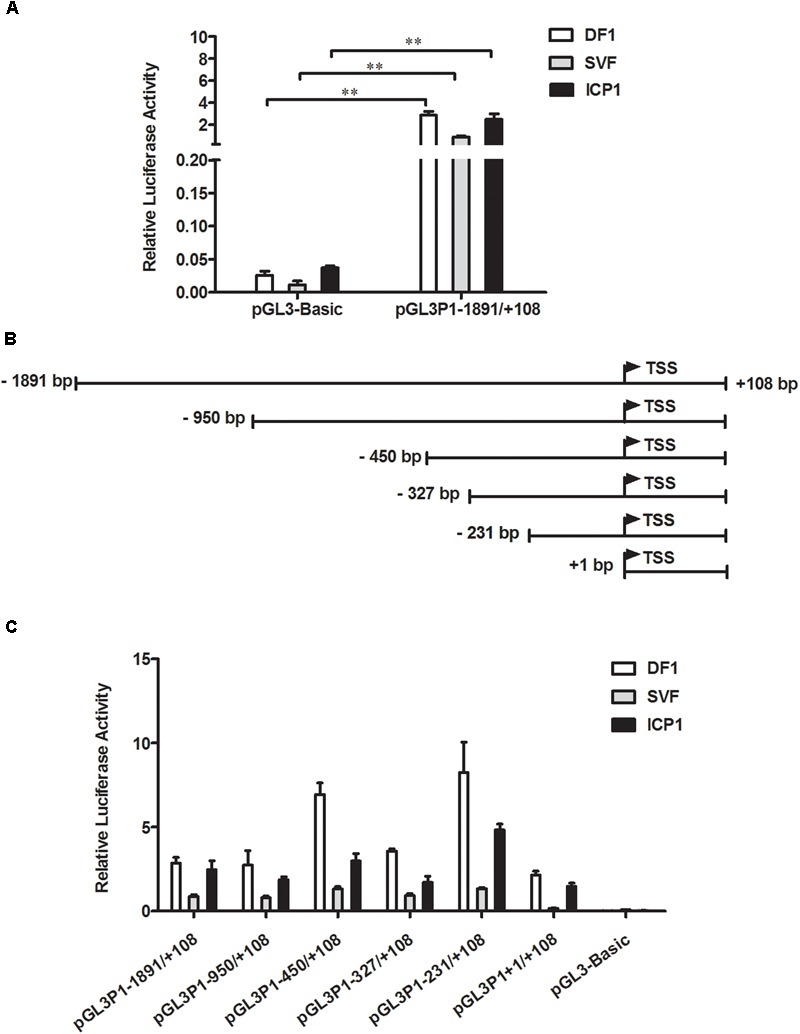
Characterization of the chicken *PPARγ* gene P1 promoter. **(A)** Luciferase activity of the P1 promoter reporter construct pGL3P1-1891/+108 in DF1, SVF, and ICP1 cells. Cells were transfected with pGL3P1-1891/+108 along with the pRL-TK Renilla luciferase vector by using Lipofectamine 2000 reagent, and luciferase activity was determined at 48 h after transfection. **(B)** Schematic diagram of the reporter constructs of the P1 promoter and its 5′ truncation mutants. The transcription start site (TSS) of chicken *PPARγ1* is represented by the bent arrow. The positions are numbered relative to the TSS. **(C)** Truncation analysis of the P1 promoter in DF1, SVF, and ICP1 cells. The indicated P1 promoter constructs and pRL-TK vector were cotransfected into DF1, SVF, and ICP1 cells. Luciferase activity was detected 48 h after cotransfection. The pRL-TK vector was used for normalization of transfection efficiency. All data represent the mean ± SE. Statistical significance was determined by Student’s *t*-test. ^∗∗^*p* < 0.01.

To understand the transcriptional regulation of the chicken *PPARγ* P1 promoter, an ∼2-kb genomic DNA fragment spanning 1,891 bp upstream and 108 bp downstream of the transcription start site of *PPARγ1* was amplified by PCR and cloned into luciferase reporter vector pGL3-Basic. The resultant construct was named pGL3P1-1891/+108. A reporter assay showed that the luciferase activities of pGL3P1-1891/+108 were 110-, 66- and 75-fold higher than those of empty pGL3-Basic vector in a chicken embryo fibroblast (DF1) cell line, chicken SVF cells and an ICP1 cell line, respectively (Figure 1A), confirming that we had cloned the active P1 promoter. The P1 promoter was active in these three different cell lines, consistent with our previous finding that *PPARγ1* was widely expressed in various chicken tissues (Duan et al., 2015).

To delineate the sequences required for the P1 promoter, we generated a total of five 5’-deletion fragments of the P1 promoter by PCR and constructed their respective reporter constructs (pGL3P1-950/+108, pGL3P1-450/+108, pGL3P1-327/+108, pGL3P1-231/+108, and pGL3P1+1/+108) (Figure 1B). All five of these P1 truncation mutants had higher luciferase activities than did the empty pGL3-Basic vector (*p* < 0.05) (Figure 1C) and displayed a similar promoter activity pattern in DF1, SVF and ICP1 cells (Figure 1C). The promoter activity of pGL3P1-1891/+108 was essentially similar to that of pGL3P1-950/+108. Progressive 5′-truncation from -950 to -450 (pGL3P1-450/+108) or from -327 to -231 (pGL3P1-231/+108) significantly increased the promoter activity compared with that of pGL3P1-950/+108 or pGL3P1-327/+108 (*p* < 0.05) (Figure 1C), indicating the presence of strong repressive elements in the P1 promoter regions from -950 to -450 and from -327 to -231 (Figure 1C). However, compared with pGL3P1-231/+108, further 5’-truncation of 231 bp (pGL3P1+1/+108) resulted in a significant decrease in the promoter activity (*p* < 0.05) (Figure 1C), indicating the presence of positive regulatory elements in the P1 promoter region from -231 to +1. Among all P1 promoter reporter constructs, pGL3P1-231/+108 exhibited the highest luciferase activity, whereas pGL3P1+1/+108 had the minimum luciferase activity that still retained basal promoter activity compared with the empty pGL3-Basic vector (*p* > 0.05) (Figure 1C), suggesting that the +1/+108 region is the core P1 promoter region.

**FIGURE 2:**
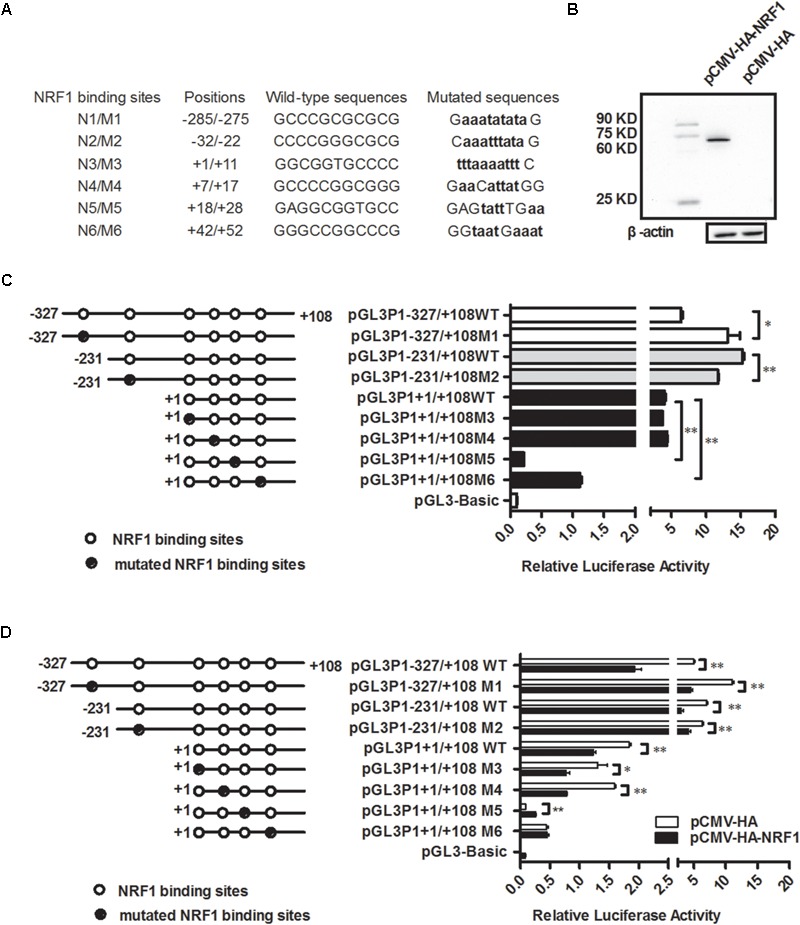
NRF1 represses P1 promoter activity. **(A)** The nucleotide sequences of the six putative NRF1 binding sites and their respective mutations in the P1 promoter. The P1 promoter mutants were created by direct DNA synthesis and subsequent cloning, and the positions of the six putative NRF1 binding sites in the P1 promoter region were numbered relative to the transcription start site (TSS). NRF1 binding sites are indicated by capital letters, and their mutated nucleotides are indicated by bold lowercase letters. **(B)** Western blot identification of chicken NRF1 expression vector (pCMV-HA-NRF1). The pCMV-HA-NRF1 or empty pCMV-HA vector was transfected into DF1 cells. The cell lysates were harvested 48 h after transfection and immunoblotted with an HA-specific antibody. **(C)** The effects of mutation of the six individual putative NRF1 binding sites on basal P1 promoter activity. The indicated P1 promoter constructs (pGL3P1-327/+108, pGL3P1-231/+108, and pGL3P1+1/+108) or indicated P1 promoter mutant constructs (pGL3P1-327/+108M1, pGL3P1-231/+108M2, pGL3P1+1/+108M3, pGL3P1+1/+108M4, pGL3P1+1/+108M5, and pGL3P1+1/+108M6), along with pRL-TK, were cotransfected into DF1 cells, and luciferase activity was determined 48 h after transfection. The open circles indicate the wild-type NRF1 binding sites, and the filled circles indicate the mutated NRF1 binding sites. All data represent mean ± SE. Statistical significance was determined by Student’s *t*-test comparing mutated versus wild-type NRF1 binding site. ^∗^*p* < 0.05, ^∗∗^*p* < 0.01. **(D)** Effects of mutation of the six individual NRF1 binding sites on NRF1-mediated inhibition of the P1 promoter. DF1 cells were cotransfected with the indicated reporter constructs along with pCMV-HA-NRF1 or empty pCMV-HA vector and pRL-TK. Luciferase activity was determined 48 h after cotransfection. All data represent the mean ± SE. Statistical significance was determined by Student’s *t*-test comparing the cotransfection of the designated reporter constructs and pCMV-HA-NRF1 versus the cotransfection of the designated reporter constructs and empty pCMV-HA vector. ^∗^*p* < 0.05, ^∗∗^*p* < 0.01.

To understand the transcriptional regulation of the P1 promoter, using JASPAR (Roy et al., 2017), we predicted the transcription factor binding sites in the -231/+108 region, which had the highest promoter activity (Figure 1C). The result showed that there were a number of putative binding sites for various transcription factors, including GATA binding protein 3 (GATA3), activating enhancer binding protein 2 (AP2), transcription factor Sp1 (Sp1) and NRF1. Of these transcription factors, NRF1 interested us. NRF1 has been known as a key transcription factor for mitochondrial biogenesis and functions (Scarpulla, 2002). Moreover, several independent lines of evidence indicated that NRF1 is implicated in lipid droplet accumulation, cell proliferation and apoptosis of the mouse preadipocyte cell line 3T3-L1 and the pathogenesis of type 2 diabetes (Cho et al., 2005; Liu et al., 2008; Tienen et al., 2010). This evidence inspired us to determine whether NRF1 regulates transcription of the P1 promoter and is involved in the regulation of adipogenesis. A detailed bioinformatics analysis showed that a total of six putative NRF1 binding sites were present in the P1 promoter region from -1891 to +108; five of them were clustered in the region -231/+108, and the other one was present at the position -285/-275 (Figure 2A). In this report, these six putative NRF1 binding sites were designated N1 (-285 to -275), N2 (-32 to -22), N3 (+1 to +11), N4 (+7 to +17), N5 (+18 to +28), and N6 (+42 to +52), relative to the transcription start site of *PPARγ1* (Figure 2A), and the mutated binding sites were referred to as M1, M2, M3, M4, M5, and M6 (Figure 2A).

### NRF1 Inhibits the P1 Promoter Activity

To test whether NRF1 regulates the P1 promoter, we initially constructed and confirmed an NRF1 expression vector, pCMV-HA-NRF1 (Figure 2B). Then, DF1 cells were transiently cotransfected with the P1 reporter constructs (pGL3P1-327/+108, pGL3P1-231/+108, or pGL3P1+1/+108) and pCMV-HA-NRF1 or pCMV-HA vector plus pRL-TK Renilla luciferase vector. The P1 reporter construct pGL3P1-327/+108 contained all six putative NRF1 binding sites, pGL3P1-231/+108 contained five of six putative NRF1 binding sites (except for N1), and pGL3P1+1/+108 contained four of these NRF1 binding sites (except for N1 and N2). A reporter assay showed that transfection with pCMV-HA-NRF1 significantly inhibited the luciferase reporter activity of these three reporters (pGL3P1-327/+108, pGL3P1-231/+108, and pGL3P1+1/+108) (*p* < 0.01) (Figure 2D), suggesting that NRF1 negatively regulates the P1 promoter.

To define which NRF1 binding sites are required for the NRF1-mediated inhibition of the P1 promoter, we mutated individual NRF1 binding sites by site-directed mutagenesis using DNA synthesis and generated their reporter constructs based on the aforementioned promoter constructs (Figures 2C,D). In brief, the mutated N1 and N2 binding site promoter reporter constructs were generated based on pGL3P1-327/+108 and pGL3P1-231/+108, respectively, and their mutant reporter constructs were designated pGL3P1-327/+108M1 and pGL3P1-231/+108M2. The mutated N3, N4, N5, and N6 binding site promoter constructs were generated based on pGL3P1+1/+108, and the resultant mutant reporter constructs were designated pGL3P1+1/+108M3, pGL3P1+1/+108M4, pGL3P1+1/+108M5, and pGL3P1+1/+108M6, respectively. First, we tested the effect of these individual NRF1 binding site mutations on the basal activity of the P1 promoter. A reporter gene assay showed that compared with their respective wild-type reporters, N1 mutation caused a 104.68% increase in basal promoter activity (*p* < 0.05) (Figure 2C), but N2 mutation led to a 23.41% reduction in P1 promoter activity (*p* < 0.01) (Figure 2C). Both N3 and N4 mutations had no effect on basal promoter activity (*p* > 0.05) (Figure 2C). In contrast, N5 and N6 mutations caused a 94.70 and 72% reduction, respectively, in basal promoter activity (*p* < 0.01) (Figure 2C). These results suggest that these NRF1 binding sites have different regulatory roles in basal P1 promoter activity.

Then, we investigated which NRF1 binding sites are required for the NRF1-mediated inhibition of the P1 promoter. Our mutation analysis showed that N2 mutation markedly reduced the inhibitory effect of NRF1 on the promoter activity of pGL3P1-231/+108 by 38.57% (*p* < 0.05) (Figure 2D). Both N5 and N6 mutations almost entirely abolished the inhibitory effect of NRF1 on the promoter activity of pGL3P1+1/+108 (Figure 2D). However, N1 mutation had no obvious effect on the inhibitory effect of NRF1 on the promoter activity of pGL3P1-327/+108 (*p* > 0.05) (Figure 2D). In contrast, N3 and N4 mutations enhanced the inhibitory effect of NRF1 on the promoter activity by 26 and 55.27%, respectively (Figure 2D). Collectively, these results suggested that the binding sites N2, N5 and N6, but not N1, N3 and N4, are required for NRF1-mediated inhibition of the P1 promoter.

### NRF1 Binds to the P1 Promoter

**FIGURE 3:**
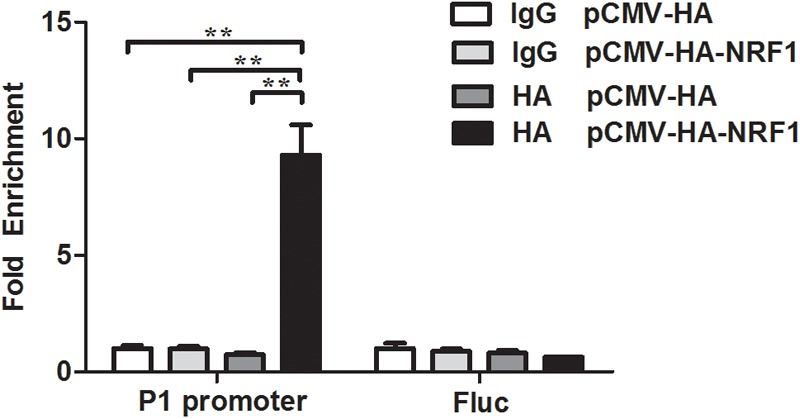
NRF1 directly binds to the P1 promoter. DF1 cells were cotransfected with the P1 promoter construct (pGL3P1-1891/+108) and either pCMV-HA-NRF1 or empty pCMV-HA vector. At 48 h after transfection, ChIP was performed with an HA-specific antibody or mouse IgG. Immunoprecipitated DNA was quantified by qPCR using two specific pairs of primers. One pair of primers for the P1 promoter region from –60 to +52 was used to determine the enrichment of NRF1 binding to P1 promoter, and the other pair of primers for the coding region of the firefly luciferase gene (*Fluc*) was used to determine the enrichment of NRF1 binding to the *Fluc* gene coding region and was used as a negative control. Non-immunoprecipitated DNA (2%) was used as an input control. Data were presented as fold enrichment over the negative control, which was prepared by cotransfection of DF1 cells with empty pCMV-HA vector and pGL3P1-1891/+108 and immunoprecipitated with mouse IgG (set to 1). All data represent the mean ± SE. Statistical significance was determined by Student’s *t*-test. ^∗∗^*p* < 0.01.

To investigate whether NRF1 directly binds to and regulates the P1 promoter, we employed a chromatin immunoprecipitation (ChIP) assay. The P1 reporter construct (pGL3P1-1891/+108) and pCMV-HA-NRF1 vector were cotransfected into DF1 cells, and ChIP was performed with an HA-specific antibody or mouse IgG (negative control). Two additional negative controls (A and B) were prepared by the cotransfection of DF1 cells with empty pCMV-HA vector and pGL3P1-1891/+108 reporter construct and immunoprecipitation with either mouse IgG (A) or HA-specific antibody (B). Enriched DNA was analyzed using quantitative PCR with a specific pair of primers (ChIP-qPCR P1 promoter, Table 1), which was designed to amplify the -60/+52 region of the P1 promoter. The ChIP-qPCR results showed that the P1 promoter fragment was significantly enriched (9-, 9- and 12-fold) in the DNA immunoprecipitated by the HA-specific antibody compared with the negative controls (mouse IgG, A and B) (*p* < 0.01) (Figure 3), but not enriched in any one of the three negative controls (mouse IgG, A and B; enrichment folds: 1:1:0.7) (*p* > 0.05) (Figure 3). The coding region of the firefly luciferase gene (Fluc) was used as a negative control in this study. As expected, the coding region of Fluc was not enriched in the DNA immunoprecipitated by HA-specific antibody compared with that by the negative controls (mouse IgG, A and B) in the ChIP-qPCR analysis using a specific pair of primers to amplify the coding region of Fluc (*p* > 0.05) (Figure 3). Taken together, these findings suggest that NRF1 can directly bind to the P1 promoter.

### Expression Patterns of NRF1 and PPARγ1 During Chicken Adipose Development

**FIGURE 4:**
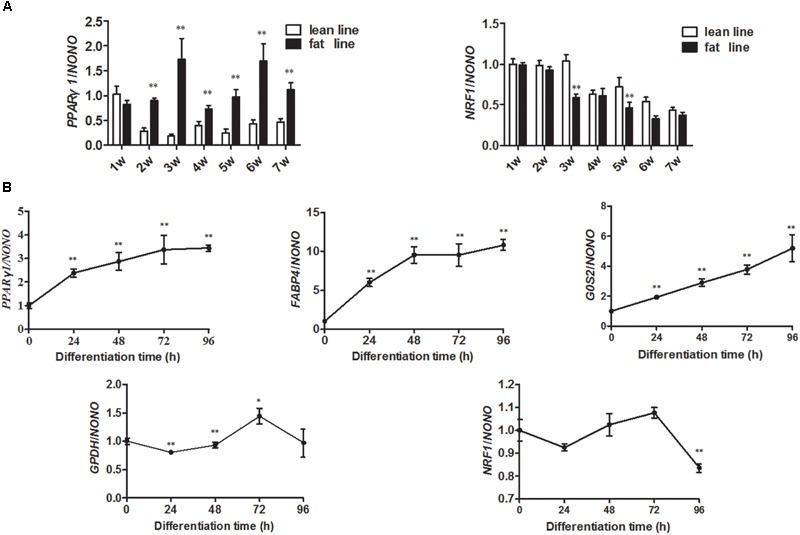
Expression levels of *PPARγ1* and *NRF1* genes during chicken abdominal adipose tissue development and ICP1 cell differentiation. **(A)** Real-time RT-PCR analysis of the expression levels of *PPARγ1* and *NRF1* in the abdominal adipose tissues of NEAUHLF (each line, *n* = 5) from 1 to 7 weeks of age. Statistical significance was determined by Student’s *t*-test comparing lean to fat lines. ^∗^*p* < 0.05, ^∗∗^*p* < 0.01. **(B)** Real-time RT-PCR analysis of the expression levels of *NRF1, PPARγ1* and adipogenic differentiation markers (*FABP4, G0S2, GPDH*, and *AdipoQ*) during differentiation of ICP1 cells. At 50% confluence, ICP1 cells were induced to differentiate by adding 160 μM sodium oleate with medium changes every 24 until 96 h of culture. The cells were harvested at 0, 24, 48, 72, and 96 h of differentiation. Chicken *NONO* was used as the internal control. The expression levels of *PPARγ1, FABP4, G0S2, GPDH*, and *NRF1* at the indicated time points are expressed relative to the expression of the respective genes in ICP1 cells at 0 h of differentiation. All data represent the mean ± SE. Statistical significance was determined by Student’s *t*-test comparing expression at the indicated time points versus 0 h of ICP1 cell differentiation. ^∗^*p* < 0.05, ^∗∗^*p* < 0.01.

To investigate whether NRF1 regulates the P1 promoter *in vivo*, we analyzed the expression patterns of chicken *NRF1* and *PPARγ1* in abdominal fat tissues from Northeast Agricultural University broiler lines divergently selected for abdominal fat content (NEAUHLF) from 1 to 7 weeks of age using real-time RT-PCR. The results showed that *PPARγ1* mRNA expression was remarkably higher in fat than in lean chicken lines from 2 to 7 weeks of age (*p* < 0.05) (Figure 4A), which was consistent with our previous study (Duan et al., 2015). Conversely, *NRF1* mRNA expression was lower in fat than in lean lines from 1 to 7 weeks of age; particularly, *NRF1* mRNA expression was significantly lower in fat than in lean chicken lines at 3 and 6 weeks of age (*p* < 0.05) (Figure 4A). Correlation analysis showed that *NRF1* and *PPARγ1* mRNA expression levels were significantly negatively correlated (Pearson’s *r* = -0.148, *p* = 0.033) from 1 to 7 weeks of age in the lean and fat lines of NEAUHLF. These expression data support our finding that NRF1 negatively regulates the P1 promoter (Figure 4A).

### NRF1 Overexpression Inhibits Chicken Adipocyte Differentiation

**FIGURE 5:**
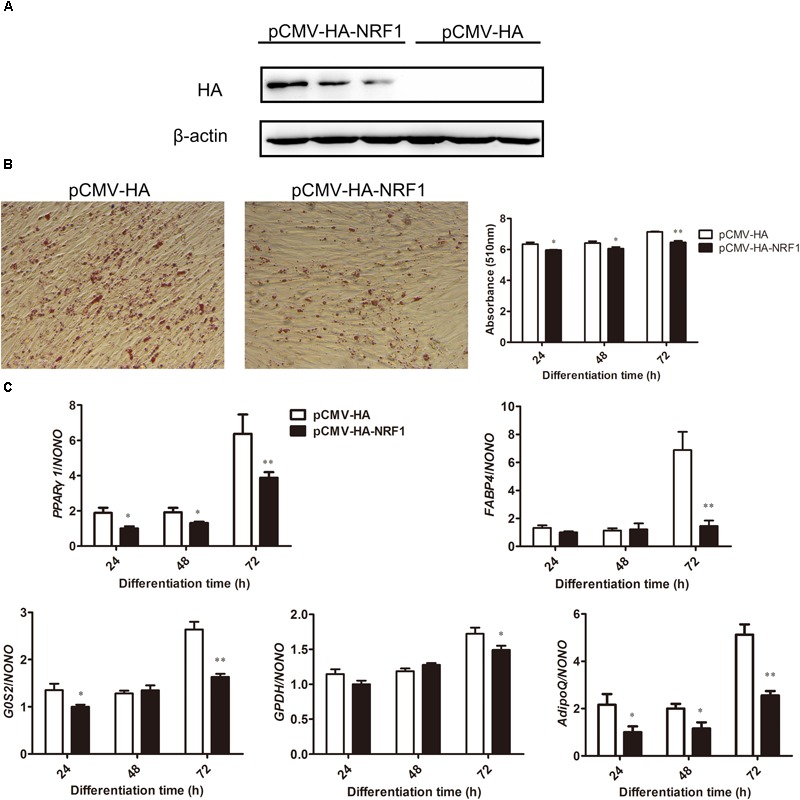
NRF1 overexpression inhibits chicken preadipocyte differentiation. **(A)** Western blot analysis of NRF1 overexpression during chicken preadipocyte differentiation. ICP1 cells were transfected with either pCMV-HA-NRF1 or empty pCMV-HA vector as a control. At 24 h after transfection, the cells were induced to differentiate by adding 160 μM sodium oleate for 24, 48, and 72 h, and the cell lysates were harvested and immunoblotted with an HA-specific antibody. **(B)** The effects of NRF1 overexpression on lipid droplet accumulation during ICP1 cell differentiation. Oil Red O staining of the ICP1 cells transfected with pCMV-HA or pCMV-HA-NRF1 was performed at 72 h of differentiation (left two panels) and its quantification at 24, 48, and 72 h (right panel). Statistical significance was determined by Student’s *t*-test comparing empty pCMV-HA vector versus pCMV-HA-NRF1 transfection. ^∗^*p* < 0.05, ^∗∗^*p* < 0.01. **(C)** Real-time RT-PCR analysis of expression levels of *PPARγ1* and adipogenic differentiation markers (*FABP4, G0S2, GPDH*, and *AdipoQ*) during the differentiation of ICP1 cells transfected with either empty pCMV-HA vector or pCMV-HA-NRF1 at the indicated times points. All data represent the mean ± SE. Statistical significance was determined by Student’s *t*-test comparing empty pCMV-HA vector versus pCMV-HA-NRF1 transfection. ^∗^*p* < 0.05, ^∗∗^*p* < 0.01.

*PPARγ* is a master regulator of adipogenesis. To test whether NRF1 is also involved in the regulation of adipogenesis, we first assayed the expression of *NRF1* and *PPARγ1* during differentiation of ICP1 cells induced by sodium oleate. Differentiation was evident as indicated by increasing expression levels of *FABP4, G0S2, GPDH*, and *AdipoQ* from 0 to 96 h after induction of differentiation (Figure 4B). The *NRF1* mRNA level remained relatively constant up to 72 h after induction of differentiation (*p* > 0.05) but significantly decreased at 96 h to a level lower than that in ICP1 cells at 0 h of differentiation (*p* < 0.01) (Figure 4B). By contrast, *PPARγ1* mRNA expression was continuously upregulated throughout the 96 h time course of differentiation (Figure 4B). Then, we examined the effect of NRF1 overexpression on chicken preadipocyte differentiation by transient transfection of pCMV-HA-NRF1 into ICP1 cells. After 24 h of transfection, the cells were induced to differentiate by sodium oleate. Western blot confirmed that the NRF1 protein was overexpressed at 24, 48, and 72 h of differentiation of ICP1 cells transfected with pCMV-HA-NRF1 compared to the cells transfected with empty pCMV-HA vector (Figure 5A).

Oil red O staining and its quantification showed that compared with the empty vector-transfected cells, NRF1 overexpression significantly decreased lipid droplet accumulation at 24, 48, and 72 h of differentiation (*p* < 0.05) (Figure 5B). Consistent with the Oil red O staining results, the expression levels of adipogenic markers *FABP4, G0S2, GPDH*, and *AdipoQ* were significantly reduced during differentiation when NRF1 was overexpressed (*p* < 0.05) (Figure 5C). These results suggest that NRF1 inhibits chicken preadipocyte differentiation. In addition, gene expression analysis showed that compared with empty vector-transfected cells, NRF1 overexpression decreased the *PPARγ1* mRNA level at 24, 48, and 72 h of differentiation (*p* < 0.05), which is consistent with our finding that NRF1 negatively regulates the P1 promoter (Figure 2D).

## Discussion

In this study, we demonstrated for the first time that NRF1 directly negatively regulates the P1 promoter of the *PPARγ* gene and inhibits chicken adipogenesis. A luciferase reporter assay demonstrated that NRF1 inhibits P1 promoter activity (Figure 2D). The site-directed mutagenesis analysis revealed that a mutation of any one of the three putative NRF1 binding sites (N2, N5, and N6) abolished NRF1-mediated inhibition of P1 promoter activity (Figure 2D). The finding that NRF1 directly bound to the P1 promoter (Figure 3) suggests that NRF1 directly regulates transcription of the P1 promoter. Negative regulation of the P1 promoter by NRF1 was supported by our finding that *PPARγ1* and *NRF1* mRNA expression levels were negatively correlated in the fat and lean chicken lines of NEAUHLF (Figure 4A) and that NRF1 overexpression significantly decreased *PPARγ1* mRNA expression during ICP1 cell differentiation (Figure 5C). Therefore, our findings indicate that NRF1 can specifically bind to the P1 promoter, resulting in decreased expression of *cPPARγ1*.

Nuclear respiratory factor 1 is an important transcription factor that regulates mitochondrial biogenesis, functioning as an activator of nuclear-encoded mitochondrial genes such as cytochrome, mitochondrial transcription factor A (TFAM) and transcription factor B proteins (TFBs) (Scarpulla, 2012; Qi and Ding, 2012; Seo et al., 2015). Recent studies demonstrated that in addition to its roles in mitochondria, NRF1 also targets several genes such as the integrin-associated protein gene (IAP), insulin-degrading enzyme gene (IDE), and proton-coupled folate transporter gene (PCFT) and is involved in the control of cell growth (Cam et al., 2004), neurite outgrowth (Chang and Huang, 2004), diabetes mellitus (DM), Alzheimer’s disease (AD) (Zhang et al., 2012), and folate transport (Gonen and Assaraf, 2010). Our present study demonstrated that NRF1 inhibits adipogenesis, providing new evidence that NRF1 can perform multiple functions.

Mitochondria play an essential role in the differentiation and maturation of adipocytes (Wilsonfritch et al., 2003; Koh et al., 2007; De Pauw et al., 2009; Kusminski and Scherer, 2012; Boudina and Graham, 2015; Liu et al., 2015). NRF1 plays important roles in regulating mitochondrial biogenesis and function (Koh et al., 2007; De Pauw et al., 2009; Kusminski and Scherer, 2012). PPARγ is a master regulator of adipogenesis, and both PPARγ1 and PPARγ2 are capable of inducing adipogenesis (Wilsonfritch et al., 2004). Like NRF1, PPARγ1 also regulates mitochondrial gene expression, biogenesis and function (Boudina and Graham, 2015; Li et al., 2016). In the present study, our results demonstrated that NRF1 directly negatively regulates the P1 promoter of the *PPARγ* gene and inhibits chicken adipogenesis (Figures 2–5). Taking into consideration all these data, we presume that NRF1 may regulate chicken adipogenesis via directly controlling the transcription of *PPARγ1* and indirectly controlling mitochondrial biogenesis and function.

In the present study, we demonstrated that NRF1 negatively regulates the P1 promoter. Considering that a transcription factor generally has multiple target genes, we cannot exclude the possibility that NRF1 inhibits chicken preadipocyte differentiation in part by directly and/or indirectly regulating the expression of other adipogenesis regulators, including the remaining *PPARγ* transcript isoforms. For a better understanding of the molecular mechanisms of NRF1 in adipogenesis, it is worth performing ChIP-seq to identify other potential targets of NRF1 that may contribute to the inhibition of chicken preadipocyte differentiation by NRF1.

In the present study, our results showed that NRF1 overexpression inhibited ICP1 cell differentiation, as demonstrated by Oil red O staining and mRNA expression of adipogenic genes (*FABP4, G0S2, GDPH*, and *AdipoQ*). Similar to our results, Tienen et al. also found that NRF1 overexpression led to less lipid accumulation in differentiated 3T3-L1 cells; however, in contrast to our results, they found that neither increased expression of adipogenesis inhibitors, such as *Delta-like kinase (DLK)* and *GATA binding protein 2* (*GATA2*), nor decreased expression of key adipogenic markers, such as *PPARγ* and *C/EBPα* (Tienen et al., 2010), was observed when NRF1 was overexpressed during the differentiation of 3T3-L1 preadipocytes, suggesting that NRF1 overexpression seems to have no effect on 3T3-L1 preadipocyte differentiation. This discrepancy may be due to species specificity and different experimental conditions (e.g., different adipogenesis inducers and different levels of NRF1 overexpression).

Excessive abdominal fat deposition within the carcass is a challenge for the broiler chicken industry (Resnyk et al., 2013, 2017), decreasing feed efficiency and reducing yield and the nutritional and commercial value of carcass parts (Resnyk et al., 2013, 2017). Our findings provide new insight into the mechanisms underlying chicken adipogenesis and excessive fat deposition. This improved understanding may be exploited in the future to control excessive fat deposition in chickens.

In conclusion, we characterized chicken *PPARγ* P1 promoter and demonstrated that NRF1 negatively regulates the *PPARγ* P1 promoter and inhibits chicken adipogenesis.

## Author Contributions

TC performed the experiments, analyzed the data, and wrote the manuscript. TX, JH, FM, YJ, XY, YC, and HL critically revised the manuscript. NW designed the experiments, provided funding support, and critically revised the manuscript.

## Conflict of Interest Statement

The authors declare that the research was conducted in the absence of any commercial or financial relationships that could be construed as a potential conflict of interest.

## Abbreviations

AdipoQ: adiponectin
FABP4: fatty acid-binding protein 4
Fluc: firefly luciferase
G0S2: G0/G1 switch 2
GPDH: glycerol-3-phosphate dehydrogenase 2
NRF1: nuclear respiratory factor 1
PPARγ: peroxisome proliferator-activated receptor-γ
